# Demographics of Head and Neck Cancer Patients: A Single Institution Experience

**DOI:** 10.7759/cureus.1418

**Published:** 2017-07-02

**Authors:** George S Stoyanov, Martina Kitanova, Deyan L Dzhenkov, Peter Ghenev, Nikolay Sapundzhiev

**Affiliations:** 1 Department of General and Clinical Pathology, Forensic Medicine and Deontology, Faculty of Medicine, Medical University – Varna “Prof. Dr. Paraskev Stoyanov”, Varna, Bulgaria; 2 Department of Neurosurgery and Ent Diseases, Division of Ent Diseases, Faculty of Medicine, Medical University – Varna “Prof. Dr. Paraskev Stoyanov”, Varna, Bulgaria

**Keywords:** head and neck cancer, descriptive analysis, statistics, demographics

## Abstract

Introduction

Head and neck cancer (HNC) comprises a diverse group of oncological entities, originating from various tissue types and organ localizations, situated in the topographical regions of the head and neck (H&N). This single institution retrospective study was aimed at establishing the HNC patient demographics and categorizing the individual incidence of H&N malignancies, regarding their organ of origin and main histopathological type.

Materials and methods

All histologically verified cases of HNC from a single tertiary referral center were reviewed in a descriptive retrospective manner. Data sampling period was 47 months.

Results

Male to female ratio of the registered HNC cases was 3.24:1. The mean age of diagnosis was 63.84 ± 12.65 years, median 65 years. The most common HNC locations include the larynx 30.37% (n = 188), lips and oral cavity 29.08% (n = 180), pharynx 20.03% (n = 124) and salivary glands 10.94% (n = 68), with other locations such as the external nose, nasal cavity and sinuses and auricle and external ear canal harboring a minority of the cases. The main histopathological groups include squamous cell carcinoma 76.74% (n = 475) and adenocarcinoma 6.14% (n = 38), with other malignant entries such as other epithelial malignancies, primary tonsillar, mucosa-associated lymphoid tissue or parenchymal lymphomas, connective tissue neoplasias, neuroendocrine and vascular malignancies diagnosed in a minority of cases.

Conclusion

Considered to be relatively rare, HNC represents a diverse group of oncological entities with individual and specific demographic characteristics. The reported single institution results appear representative of the national incidence and characteristics of HNC.

## Introduction

Head and neck cancer (HNC) comprises a diverse group of oncological entities, originating from various tissue types and organ localizations all situated in the topographical region of the head and neck (H&N) [1-4]. The H&N region is the sixth most often site for malignancies [5-6]. It is also one of the few medical fields allowing for an uncomplicated endoscopy and diagnostic biopsy through a natural orifice in almost all cases, thus allowing detailed pretreatment diagnostic and staging [1-4, 7-8].

The reported worldwide incidence of HNC is approximately 3% of all cancer cases, with males being affected in nearly 90% of all cases and epithelial neoplasms representing more than 85% of all cancer types [2-4, 9-12]. The incidence of HNC in developing countries, however, is higher due to the prevalence of risk factors such as smoking and alcohol consumption. HNC incidence in Bulgaria as per the latest data published by the National Oncological Registry for 2013 reports 1,626 (4.42%) HNC cases out of 36,825 newly reported cancer cases, for a total population of 7,245,677 people [13].

The design of this retrospective, single institution study was aimed at establishing the HNC patient demographics and categorizing the individual incidence of H&N malignancies in a descriptive manner, with regard to their organ of origin and main histopathological type.

## Materials and methods

All histologically verified cases of HNC from the central pathological archive of a single medical institution – St. Marina University Hospital, Varna, Bulgaria, a tertiary referral center – were reviewed in a retrospective manner. The overall period of data sampling was 47 months (September 2012–July 2016).

The patient identification was based on the unique hospital ID number and Unified Citizen Number. Therefore, a "case" was determined as a patient with HNC, histologically verified in our hospital and not as an individual HNC biopsy.

The study cohort comprised 619 patients, with the total number of biopsy specimens reviewed exceeding 1,500, as many patients had multiple biopsies in the different stages of their treatment and follow-up.

The establishment of these criteria evaded statistical blurring from patients biopsied or operated more than once in the set timeframe. Recurrent HNC in the set timeframe was also excluded from the statistical analysis. Recurrent HNC however, diagnosed prior to the start of the collection of the data was included as an HNC case. All cases of metastatic disease in the H&N region were excluded from the statistical analysis, due to the study being aimed only at primary HNC. Locally invasive forms of skin cancer, directly associated with an organ of interest, were also included into the cohort.

Due to the criteria of histological verification of the type of HNC in our institution, patients treated conservatively in our institution for HNC, in wards such as oncology, radiotherapy and palliative care, with a tumor histologically verified in other medical institution, were excluded from the statistical analysis.

## Results

**Figure 1:**
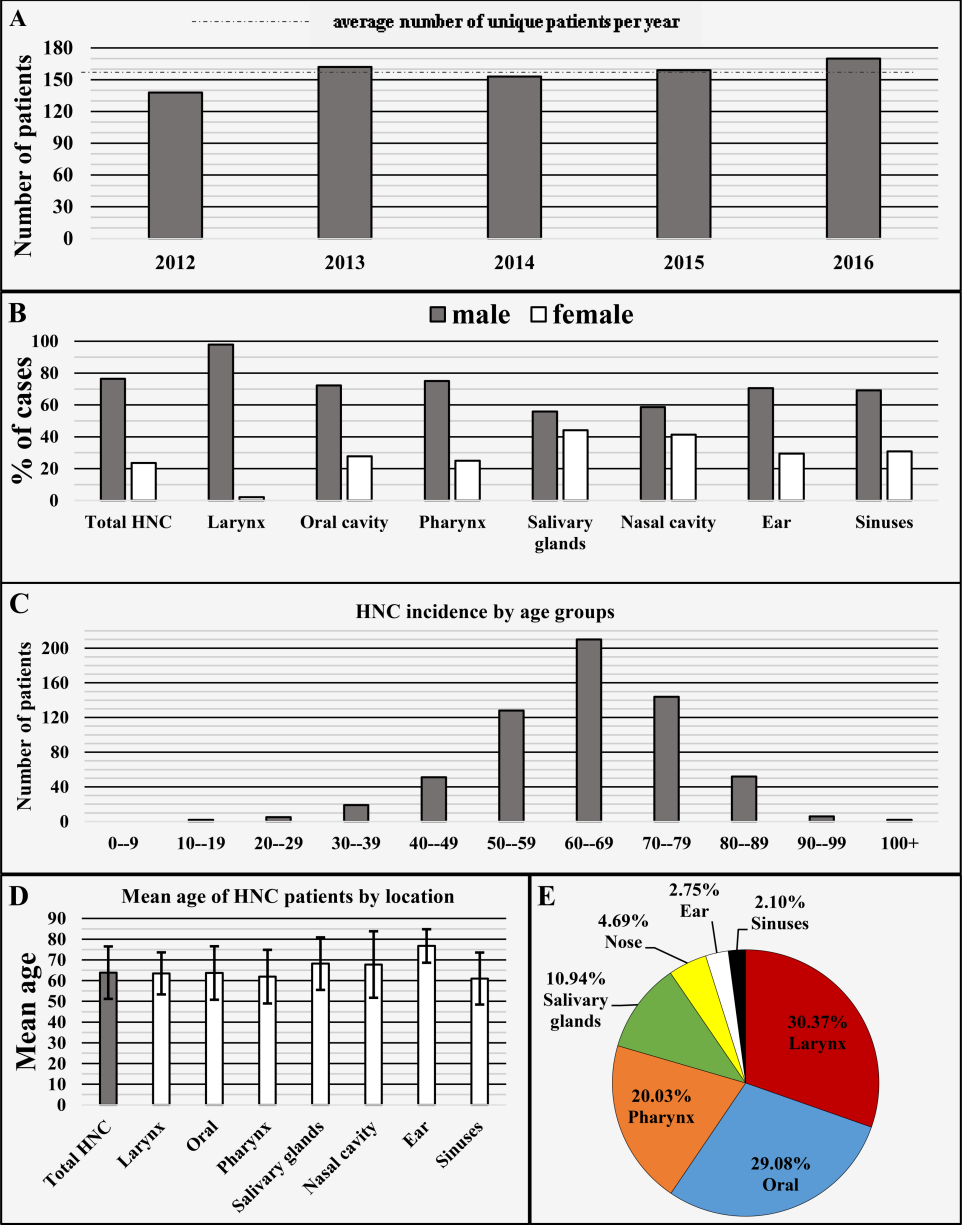
Head and neck cancer by distribution of cases on a yearly basis (A), localizations with gender ratio (B), incidence by age group (C), mean age (D) and general distribution by major localizations (E). HNC: Head and neck cancer.

A total of 619 individual HNC cases were registered in the set timeframe across 618 patients. The only patient who had two cases of HNC registered was a 46-year-old female patient first diagnosed with squamous cell carcinoma (SCC), who later developed a mucosa-associated lymphoid tissue (MALT) lymphoma in a different H&N location. These types of synchronous and metachronous tumors are a rare citing worldwide [14]. The yearly distribution of cases, calculated on a patient-yearly basis, was n = 138 individual cases for 2012, n = 162 for 2013, n = 153 for 2014, n = 159 for 2015 and n = 170 for 2016, resulting in an average of 156 unique HNC cases per year (Figure 1A).

Of all the registered HNC cases 76.41% (n = 473) were diagnosed in males and 23.59% (n = 146) in females, with male to female ratio of 3.24:1 (Figure 1B). The mean age of diagnosis was 63.84 ± 12.65 years, median 65 years, with the youngest patient diagnosed being 14 years old and the oldest 103 years of age at the time of diagnosis (Figure 1C-1D).

The primary localizations included the larynx 30.37% (n = 188) cases, lips and oral cavity 29.08% (n = 180), pharynx 20.03% (n = 124), salivary glands 10.94% (n = 68), external nose and nasal cavity 4.69% (n = 29), auricle and external ear canal 2.75% (n = 17) and sinuses 2.1% (n = 13) (Figure 1E).

**Figure 2:**
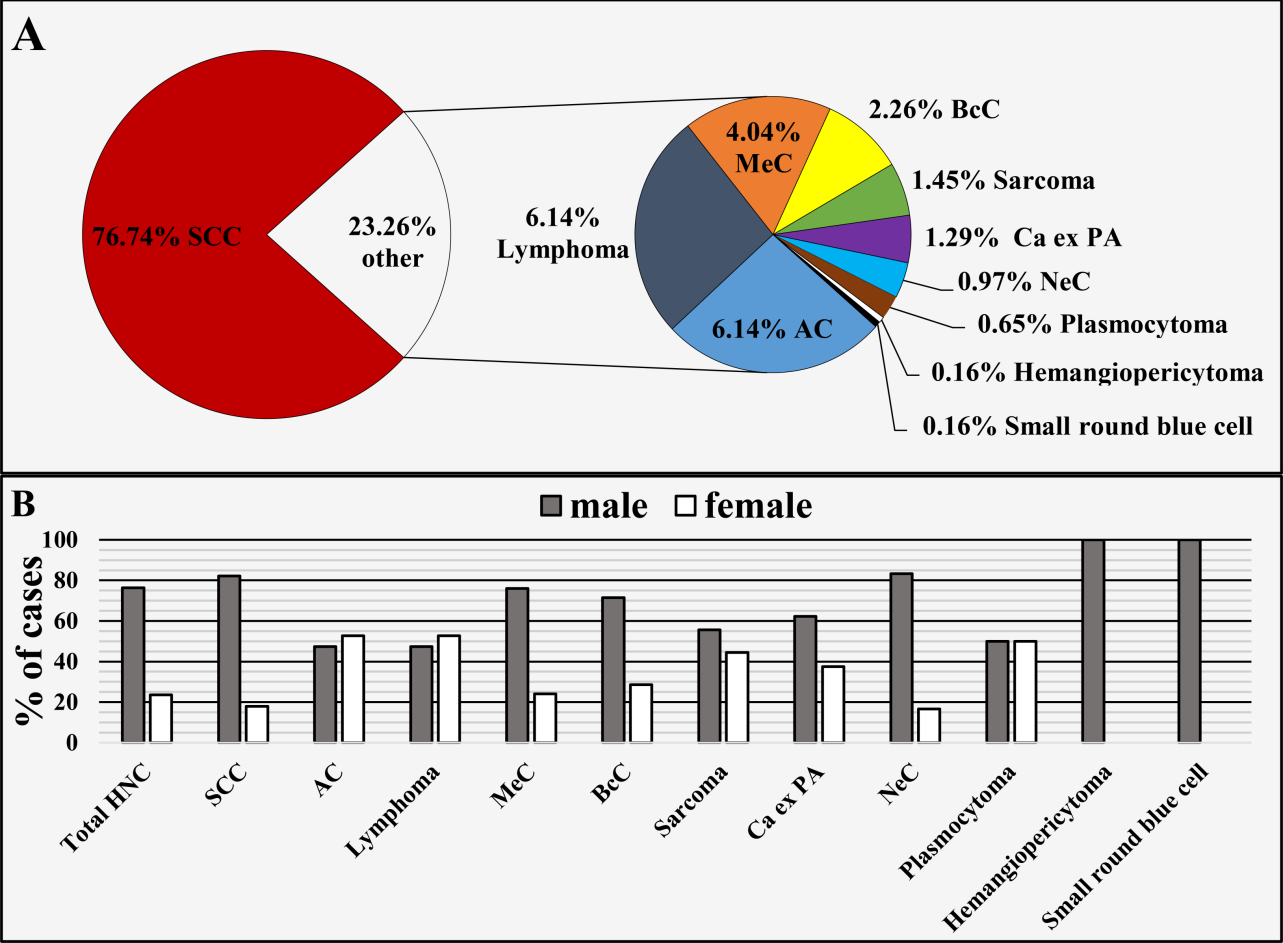
Head and neck cancer by main histopathological type (A) and gender ratio of each histopathological type (B). HNC: Head and neck cancer; SCC: Squamous cell carcinoma; AC: Adenocarcinoma; MeC: Mucoepidermoid carcinoma; BcC: Basocellular carcinoma; Ca ex PA: Carcinoma ex pleomorphic adenoma; NeC: Neuroendocrine carcinoma.

Regarding the main histopathological groups SCC comprised 76.74% (n = 475), adenocarcinoma (AC) 6.14% (n = 38), primary tonsillar, MALT or parenchymal lymphomas 6.14% (n = 38), mucoepidermoid carcinoma (MeC) 4.04% (n = 25), basocellular carcinoma (BcC) 2.26% (n = 14), different types of sarcoma 1.45% (n = 9), carcinoma ex pleomorphic adenoma (Ca ex PA) 1.29% (n = 8), neuroendocrine carcinoma (NeC) 0.97% (n = 6), plasmocytoma 0.65% (n = 4), small round blue cell tumor 0.16% (n = 1) and hemangiopericytoma 0.16% (n = 1) (Figure 2A). The gender ratio of each individual histopathological group is shown in Figure 2B.

The individual HNC entities by their localization, main histological type, age and gender of the patient and the yearly registered cases with regards to their localizations code in the first revision of the International Classification of Diseases for Oncology 3 (ICD-O 3) are as follows [15]:

Larynx and cervical region of the trachea (ICD-O 3: C32.0-C33.9)

**Figure 3:**
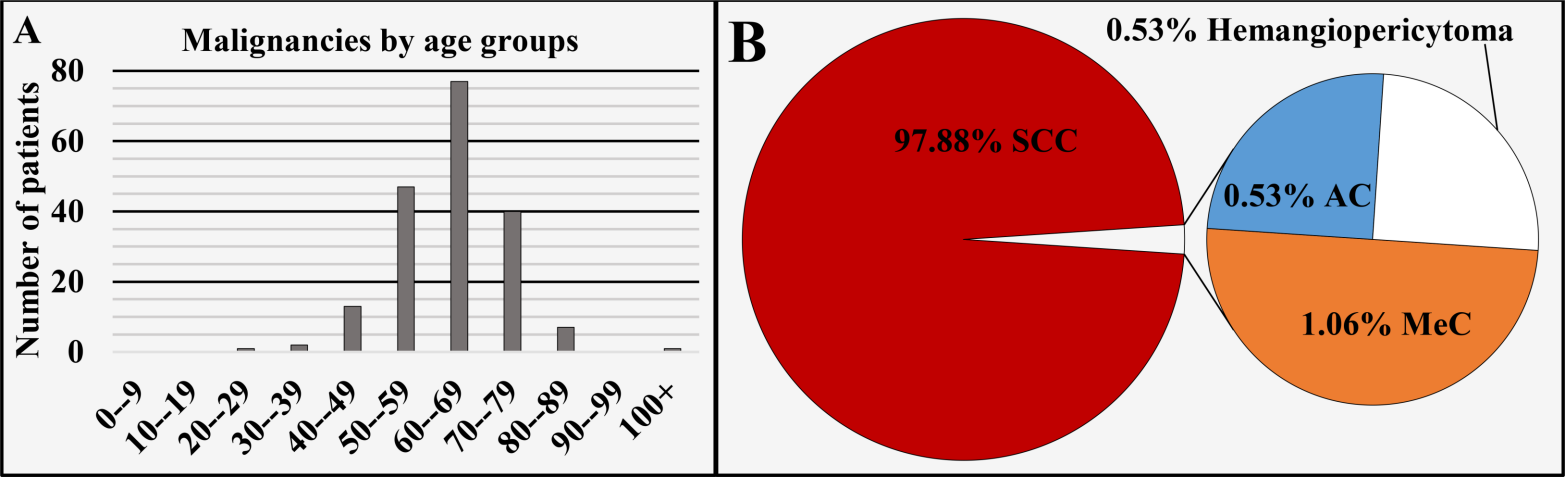
Malignancies of the larynx and cervical region of the trachea by age group (A) and main histopathological type (B). SCC: Squamous cell carcinoma; AC: Adenocarcinoma; MeC: Mucoepidermoid carcinoma.

Of all 188 cases registered in this region, 97.88% (n = 184) were registered in the larynx and 2.12% (n = 4) were registered in the cervical region of the trachea. From all the cases, 92.55% (n = 174) were diagnosed in males and 7.45% (n = 14) in females, with a male to female ratio of 12.43:1 (Figure 1B), mean age of diagnosis of 63.48 ± 10.14 years, median 64 years (Figure 1D and Figure 3A). The different oncological entities were represented with 97.88% (n = 184) SCC, 1.06% (n = 2) MeC, 0.53% (n = 1) AC and 0.53% (n = 1) hemangiopericytoma (Figure 3B). A total of 6.38% (n = 12) of the cases were registered in the non-invasive stage of carcinoma in situ.

Lips and oral cavity (ICD-O 3: C00.0-C08.9)

**Figure 4:**
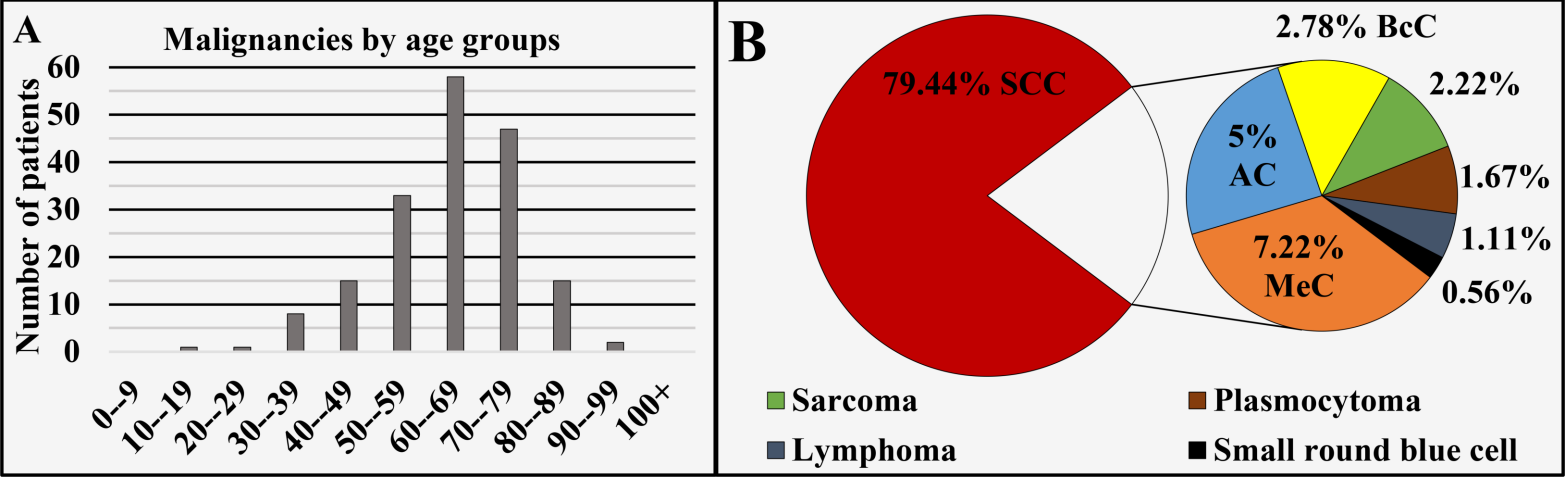
Malignancies of the lip and oral cavity by age group (A) and main histopathological type (B). SCC: Squamous cell carcinoma; AC: Adenocarcinoma; MeC: Mucoepidermoid carcinoma; BcC: Basocellular carcinoma.

Of all 180 cases registered in this region, 72.22% (n = 130) were diagnosed in males and 27.78% (n = 50) in females, with a male to female ratio of 2.6:1 (Figure 1B), mean age of diagnosis of 63.67 ± 12.9 years, median 65 years (Figure 1D and Figure 4A). The different oncological entities were represented with 79.44% (n = 143) SCC, 7.22% (n = 13) MeC, 5% (n = 9) AC, 2.78% (n = 5) BcC, 2.22% (n = 4) sarcoma, 1.67% (n = 3) plasmocytoma, 1.11% (n = 2) lymphoma and 0.56% (n = 1) embryonal undifferentiated small round blue cell tumor (Figure 4B). A total of 2.22% (n = 4) of the cases were registered in the non-invasive stage of carcinoma in situ.

Pharynx (ICD-O 3: C0.9-C14.2)

**Figure 5:**
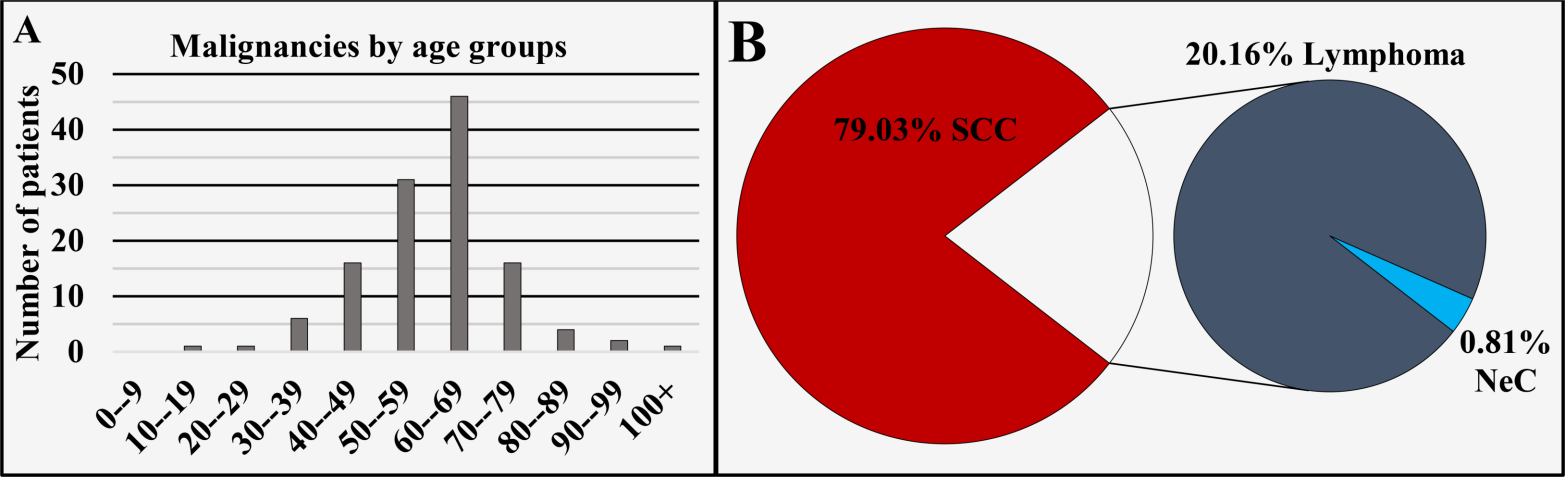
Malignancies of the pharynx by age group (A) and main histopathological type (B). SCC: Squamous cell carcinoma; NeC: Neuroendocrine carcinoma.

Of all 124 cases registered in this region, 75% (n = 93) were diagnosed in males and 25% (n = 31) in females, with a male to female ratio of 3:1 (Figure 1B), mean age of diagnosis of 61.92 ± 12.94 years, median 61 (Figure 1D and Figure 5A). The different oncological entities were represented with 79.03% (n = 98) SCC, 20.16% (n = 25) lymphoma and 0.81% (n = 1) NeC (Figure 5B). Malignant formations were isolated from all three portions of the pharynx. A total of 0.81% (n = 1) of the cases were registered in the non-invasive stage of carcinoma in situ.

Salivary glands (ICD-O 3: C08.0-C08.9)

**Figure 6:**
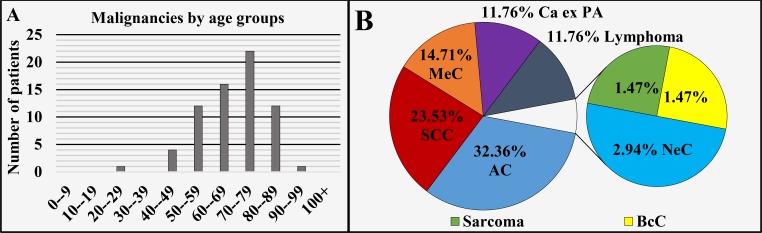
Malignancies of the salivary glands by age group (A) and main histopathological type (B). SCC: Squamous cell carcinoma; AC: Adenocarcinoma; MeC: Mucoepidermoid carcinoma; BcC: Basocellular carcinoma; Ca ex PA: Carcinoma ex pleomorphic adenoma; NeC: Neuroendocrine carcinoma.

Of all 68 cases registered in this region, 55.88% (n = 38) were diagnosed in males and 44.12% (n = 30) in females, with a male to female ratio of 1.27:1 (Figure 1B), mean age of diagnosis of 68.19 ± 12.68 years, median 70 years (Figure 1D and Figure 6A). The different oncological entities were represented with 32.36% (n = 22) AC, 23.53% (n = 16) locally invasive SCC, 14.71% (n = 10) MeC, 11.76% (n = 8) Ca ex PA, 11.76% (n = 8) lymphoma, 2.94% (n = 2) NeC, 1.47% (n = 1) sarcoma and 1.47% (n = 1) locally invasive BcC (Figure 6B).

External nose and nasal cavity (ICD-O 3: C30.0 and C44.3)

**Figure 7:**
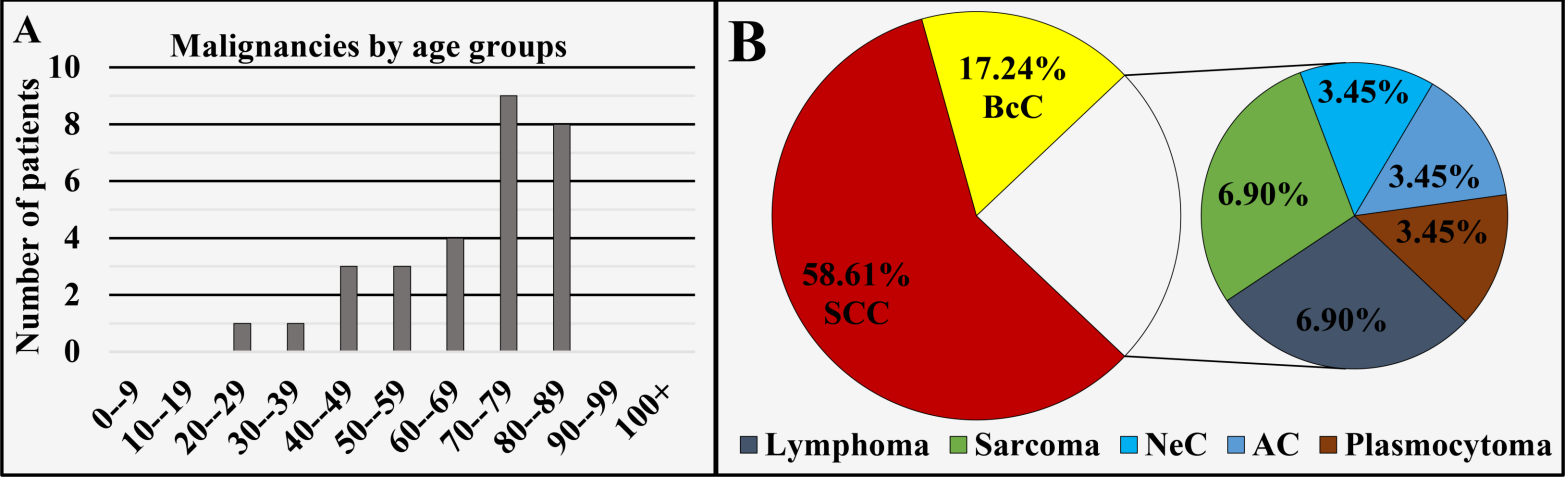
Malignancies of the external nose and nasal cavity by age group (A) and main histopathological type (B). SCC: Squamous cell carcinoma; AC: Adenocarcinoma; BcC: Basocellular carcinoma; NeC: Neuroendocrine carcinoma.

Of all 29 cases registered in this region, 58.61% (n = 17) were diagnosed in males and 41.39% (n = 12) in females, with a male to female ratio of 1.42:1 (Figure 1B), mean age of diagnosis of 67.76 ± 16.04 years, median 72 years (Figure 1D and Figure 7A). The different oncological entities were represented with 58.61% (n = 17) SCC, 17.24% (n = 5) BcC, 6.9% (n = 2) lymphoma, 6.9% (n = 2) sarcoma, 3.45% (n = 1) NeC, 3.45% (n = 1) AC and 3.45% (n = 1) plasmocytoma (Figure 7B).

Auricle and external ear canal (ICD-O 3: C44.2 and C44.3)

**Figure 8:**
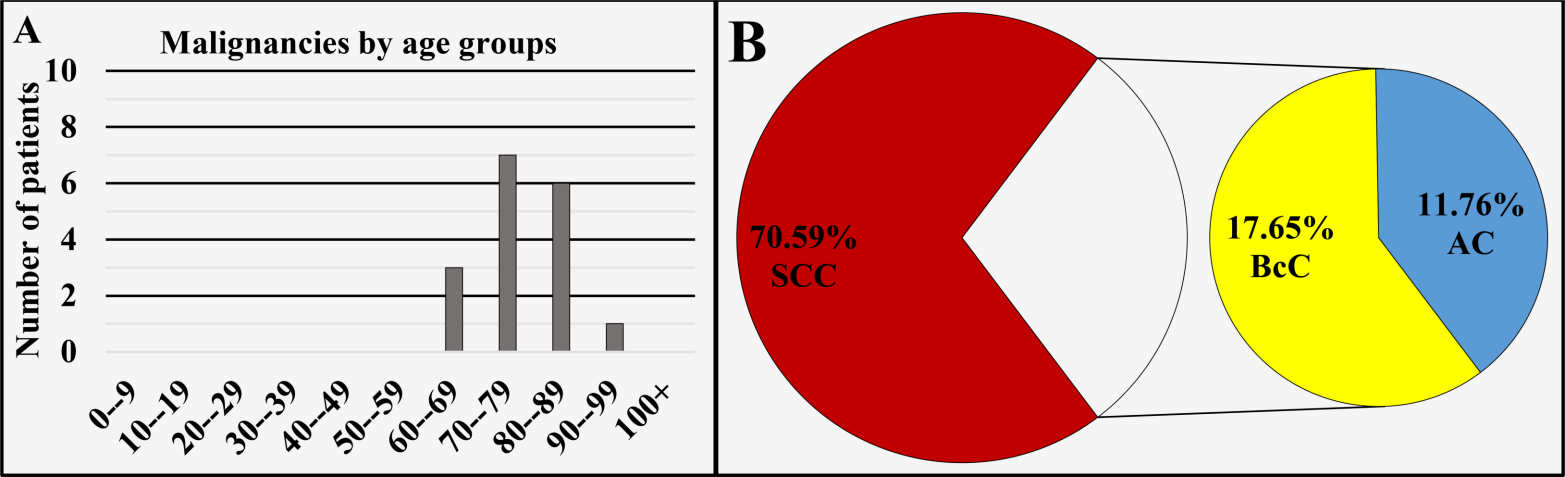
Malignancies of the auricle and external ear canal by age group (A) and main histopathological type (B). SCC: Squamous cell carcinoma; AC: Adenocarcinoma; BcC: Basocellular carcinoma.

Of all 17 cases registered in this region, 70.59% (n = 12) cases were diagnosed in males and 29.41% (n = 5) in females, with a male to female ratio of 2.4:1 (Figure 1B), mean age of diagnosis of 76.71 ± 8.08 years, median 75 years (Figure 1D and Figure 8A). The different oncological entities were represented with 70.59% (n = 12) SCC, 17.65% (n = 3) BcC and 11.76% (n = 2) AC (Figure 8B).

Sinuses (ICD-O 3: C31.0-C31.9)

**Figure 9:**
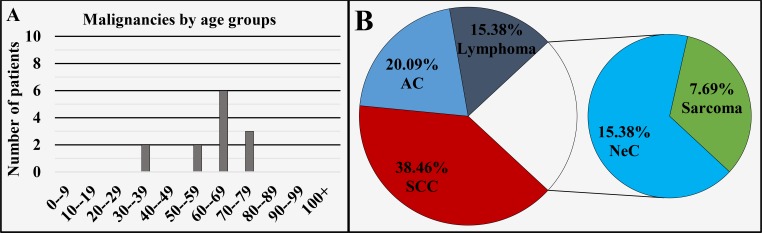
Malignancies of the sinuses by age group (A) and main histopathological type (B). SCC: Squamous cell carcinoma; AC: Adenocarcinoma; NeC: Neuroendocrine carcinoma.

Of all 13 cases registered in this region, 69.23% (n = 9) cases were diagnosed in males and 30.77% (n = 4) in females, with a male to female ratio of 2.25:1 (Figure 1B), mean age of diagnosis of 61 ± 12.54 years, median 63 years (Figure 1D and Figure 9A). The different oncological entities were represented with 38.46% (n = 5) SCC, 23.09% (n = 3) AC, 15.38% (n = 2) lymphoma, 15.38% (n = 2) NeC and 7.69% (n = 1) sarcoma (Figure 9B).

## Discussion

HNC presents an important medical problem. This anatomical region is the sixth most often localization of malignancies. In up to 90% of all these cases, the histopathological type reported is SCC [5-6, 16]. The most pronounced risk factors are tobacco and alcohol consumption [17-19]. SCC, however, is only one of the many histopathological groups of cancers that can arise in the H&N area. Many other cell types in the region can undergo malignant transformation and give rise to malignant growth, requiring different clinico-pathological criteria for grading and staging, with different treatment strategies and prognosis for each different tumor type.

In our report, exclusions from the list of presented data are entities from some organs situated in the H&N area, the malignant tumors, which fall into a different category, due to their organ specifics and characteristics. All cases of metastatic HNC were excluded from the study cohorts, as we aimed only at establishing the incidence and demographics only of primary cases. Other omitted entities include thyroid and parathyroid malignancies, which fall into the category of endocrine neoplasms. Lymph nodes, which fall into the category of hematological malignancies, if not metastatic and skin malignancies, not directly associated with an organ of interest, which fall into the category of skin cancer. More specific cancer types found in the H&N region were also excluded, such as odontological and bone malignancies, which fall into separate groups of their own and central nervous system and ocular malignancies, falling into the categories of central nervous system neoplasms and ocular malignancies, respectively. Despite this, some statistical analysis includes these tumors, based purely on their topographical localizations [3, 20].

We observed some variation in the incidence of HNC by localization of the primary malignancy on yearly basis, which we would attribute to non-medical reasons, such as the natural shift in the referral preferences of the primary and secondary centers, the preferences of the patients, as well as changes in the capacities of related departments in our institution. Despite all this, the total incidence of HNC remains near unchanged in the separate years and the mean age of diagnosis is nearly identical across most localizations, as reported by other working groups [21]. However, the retrospective nature of the study and its short timeframe, combined with it being carried only in one medical center make it hard to establish a tendency for dynamic in HNC demographics, location and main histopathological site, which could be noted on a more general scale as in nationwide surveys [13].

The reported figures for gender ratio between localizations show a lower male to female ratio than the worldwide data, but higher than the figures reported in the developed countries. We observed a lower percentage of SCC than that of the global population [2-4, 21].

The frequencies observed at our institution correspond to the overall proportions for the different primary localizations and histological types as compared to the latest published national oncology reports, where the HNC rates follow a nearly identical rate and statistical curves as the currently reported values, for the respective years [13, 22-24].

With the widespread prevalence of the major known risk factors for developing HNC across the Bulgarian population, the results seem to be representative and are a critical reflection of the level of medical care in the country and further need to increase the quality and volume of activities in prevention politics [19, 25-28].

Our retrospective analysis does not include some casuistically rare types of HNC, which can be registered in the H&N region, due to their extreme rarity and lack of patients with such diagnoses in the period of data sampling. These rare tumor types are very rarely included in any statistical analysis and are most often just reported as individual cases [2-4, 29].

Some HNC patients may have a secondary malignancy in another anatomical region or a metastatic HNC formation, however, our study design was aimed only at the H&N region and primary HNC. Only one patient with a second type of HNC in the set timeframe was reported. Our study design appears quite insensitive to such data and this single case observed is not representative for synchronous malignancies. Anyhow, data on synchronous and metachronous HNC is rarely reported [14].

## Conclusions

HNC is a relatively rare subset of cancer in Bulgaria, accounting for just above 4% of cancer cases. It, however, represents a major and likely increasing medical and social problem. Our data offers valuable statistical insight for the demographics of HNC patient in Bulgaria, while comparing the localizations of interest, main histopathological groups and the rate of yearly diagnosed cases, for the first time in such depth. Future public health efforts at primary prevention through reducing the modifiable risk factors should be encouraged.
